# Varying efficacy of intermittent preventive treatment for malaria in infants in two similar trials: public health implications

**DOI:** 10.1186/1475-2875-6-132

**Published:** 2007-09-26

**Authors:** Clara Menendez, David Schellenberg, Eusebio Macete, Pedro Aide, Elizeus Kahigwa, Sergi Sanz, John J Aponte, Jahit Sacarlal, Hassan Mshinda, Marcel Tanner, Pedro L Alonso

**Affiliations:** 1 Barcelona Center for International Health Research (CRESIB), Hospital Clinic, Institut d'Investigacions Biomedicas August Pi i Sunyer (IDIBAPS), Universitat de Barcelona, Spain; 2 Manhiça Health Research Center, Manhiça (CISM), Mozambique; 3 National Directorate of Health, Maputo, Mozambique; 4 National Institute of Health, Mozambique; 5 Ifakara Health Research and Development Centre, Ifakara, Tanzania; 6 Swiss Tropical Institute, Basel, Switzerland; 7 World Health Organisation- Country Office, Dar es Salaam, Tanzania; 8 London School of Hygiene and Tropical Medicine, (LSHTM), UK

## Abstract

**Background:**

Intermittent preventive treatment (IPTi) with sulphadoxine-pyrimethamine (SP) in infants resulted in different estimates of clinical malaria protection in two trials that used the same protocol in Ifakara, Tanzania, and Manhiça, Mozambique. Understanding the reasons for the discrepant results will help to elucidate the action mechanism of this intervention, which is essential for rational policy formulation.

**Methods:**

A comparative analysis of two IPTi trials that used the same study design, follow-up, intervention, procedures and assessment of outcomes, in Tanzania and Mozambique was undertaken. Children were randomised to receive either SP or placebo administered 3 times alongside routine vaccinations delivered through the Expanded Program on Immunisation (EPI). Characteristics of the two areas and efficacy on clinical malaria after each dose were compared.

**Results:**

The most relevant difference was in ITN's use ; 68% in Ifakara and zero in Manhiça. In Ifakara, IPTi was associated with a 53% (95% CI 14.0; 74.1) reduction in the risk of clinical malaria between the second and the third dose; during the same period there was no significant effect in Manhiça. Similarly, protection against malaria episodes was maintained in Ifakara during 6 months after dose 3, but no effect of IPTi was observed in Manhiça.

**Conclusion:**

The high ITN coverage in Ifakara is the most likely explanation for the difference in IPTi efficacy on clinical malaria. Combination of IPTi and ITNs may be the most cost-effective tool for malaria control currently available, and needs to be explored in current and future studies.

**Trial Registration:**

Manhiça study registration number: NCT00209795

Ifakara study registration number: NCT88523834

## Introduction

Despite increased attention to malaria control by donors, researchers, clinicians, and communities, malaria continues to exact an intolerable toll, particularly in sub-Saharan Africa. The development of new tools such as combination of drug therapies and insecticide treated nets (ITNs) have offered hope, but their impact has been limited by low implementation and logistical and financial constraints. No single tool currently exists that can drastically reduce the malaria burden. Given this reality, and while awaiting new technologies, the malaria community must reexamine available data and interventions to look for creative and synergistic control strategies.

It is well documented and accepted that the burden of malaria falls greatly on young children and infants [1]. Even in low/moderate malaria transmission settings, where older children suffer the most malaria episodes, infants have the highest fatality rate [2]. Finding cost-effective and affordable approaches to deliver malaria control interventions to infants is a public heath priority, especially since adequate control may be followed by important reductions in mortality for infants as well as young children [3]. The Expanded Program on Immunization (EPI) is the only available scheme that involves regular contact between the population at risk and the health system, even in places with very limited access to services. The intermittent preventive treatment in infants (IPTi) consists of administering a treatment dose of an antimalarial drug at predetermined intervals regardless of the presence of parasitaemia or symptoms. Through the EPI, it has the potential to become a cost-effective strategy.

IPTi with sulphadoxine-pyrimethamine (SP) has been shown to significantly reduce malaria episodes in randomized trials carried out in Tanzania, and more recently in Ghana and Mozambique [4-6]. The effect of one treatment dose of SP can last as long as 60 days [7]. Thus, the administration of SP coinciding with immunizations through the EPI scheme could provide a period of suppressive prophylaxis that retains some beneficial effects of regular chemoprophylaxis without compromising the development of malaria immunity [8].

Despite the positive results for efficacy and safety, the determinants and underlying protection mechanisms of IPTi are not yet clear. The analysis of differences between clinical trials that use similar designs could provide insight into the potential determinants and possible underlying protection mechanisms, as well as facilitate planning for future policy recommendations. We had the unique opportunity to compare two very similar IPTi trials in two malaria endemic countries in Eastern and Southern Africa. We present results from a comparative analysis of the protective efficacy of IPTi, and examine the factors that may explain the different protection levels achieved [4,6]. The goal is that this information will help garner future research and guide decision making about the most appropriate role of IPTI in malaria control.

## Methods

### Study area and population

The Tanzanian study was based in Ifakara town, Kilombero District, in rural Tanzania and is described in detail elsewhere [9]. Malaria transmission is perennial with two rainy seasons and a cool, dry season from July to September. *Plasmodium falciparum *malaria transmission has been intense in the area. The estimated mean annual entomological inoculation rate (EIR) was 300 bites/person/year in the late 1980's and early 1990's in villages surrounding Ifakara town [10]. However, in the last years, malaria transmission in the semi-urban area of Ifakara decreased [2,9] to an overall EIR of 29 infective bites per person per year [11]. *Anopheles gambiae*, and to a lesser extent *Anopheles funestus*, are the main vectors. The Ifakara population was estimated to be 55.000 people.

In Ifakara, SP was not associated with any late treatment failures, had a in vivo parasitological sensitivity of 69% at day 14 [12], and was the nationally recommended first line treatment for malaria during the study duration. Compliance to routine EPI vaccinations was high; 92% of children received three doses of DTP/OPV and 80% received measles. HIV seroprevalence at antenatal visits was 6.7% in 1998 [13], The use of insecticide treated nets (ITNs) was about 70%. The prevalence of haemoglobin AS genotype in the study children was 12% [4].

In Mozambique the trial was conducted in Manhiça town, Manhiça District, in southern Mozambique. The characteristics of the area have been described in detail elsewhere [14]. The climate is subtropical with a warm and rainy season from November to April, and a cool and dry season during the rest of the year. Perennial malaria transmission with marked seasonality is mostly due to *P. falciparum*. *Anopheles funestus *is the main vector and the EIR for 2002 was 38. The population under demographic surveillance was about 70.000 people.

During the Mozambique study, first line treatment of uncomplicated malaria changed from chloroquine to amodiaquine plus SP Most recent data from 2001 on the efficacy of SP in this area showed a combined therapeutic efficacy rate of 83% of children treated, with an *in vivo *parasitological sensitivity of 78.6% at day 14 [15]. Compliance with EPI vaccines was very high, more than 95% of children received all three doses of DTP/polio/Hepatitis B and more than 85% received measles. HIV seroprevalence in antenatal women was 19% in 2003, (Berenguera et al submitted) and ITN use was zero; only 15% of children used non-treated nets [6]. No individuals homozygous for sickle cell or carriers have been found in the area through community and hospital based surveys (Menendez unpublished data).

### Study design

**Figure 1:**
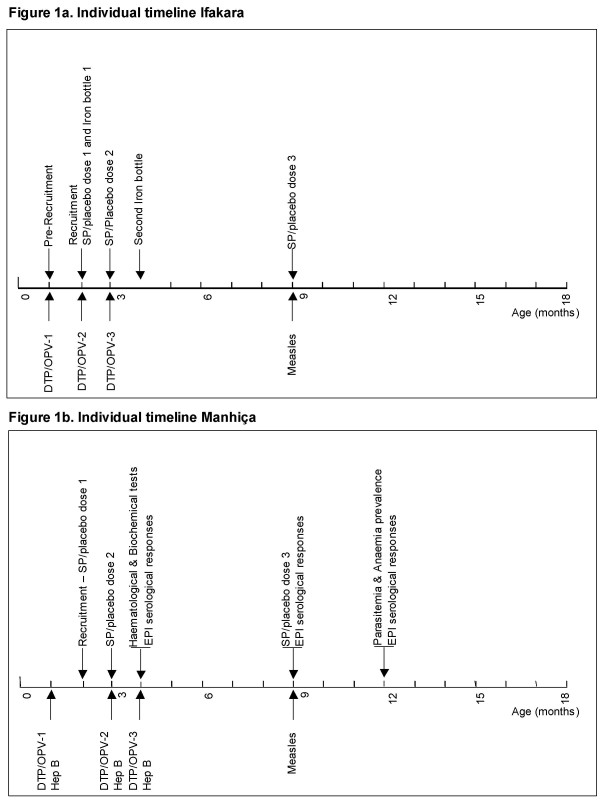
a. Individual timeline Ifakara. b. Individual timeline Manhiça.

Detailed descriptions of the Ifakara [4] and Manhica trials are provided elsewhere [6]. Briefly, in Ifakara infants were recruited at the MCH clinic immediately after receiving dose 2 of DTP/OPV between August 1999 and April 2000. IPTi was given at ages 2, 3, and 9 months alongside routine EPI vaccinations (Figure 1a). In Manhiça, children were recruited from those attending the EPI clinic to receive dose 2 of DTP/OPV/Hep B between September 2002 and February 2004 (Figure 1b). Identical randomization, blinding, treatment concealment and allocation procedures and inclusion and exclusion criteria were followed in each study. Tablets of SP and placebo (consisting of lactose and maize starch) were identical in shape and color and stored in bottles labeled only with a single treatment identification letter by investigators not involved in the studies. Placebo and SP tablets were provided by the same manufacturer (Hoffman La Roche, Basel, Switzerland). Doses of SP/placebo were administered by a health assistant according to body weight (< 5 kg-1/4 tablet, 5–10 kg-1/2 tablet, > 10 kg-1 tablet), crushed and mixed with water on a tablespoon.

### Follow-up

Assessment of outcomes was similar in both studies. Comparable round-the-clock hospital-based clinical surveillance systems were operating in both sites; at each consultation a detailed standardized questionnaire was completed to document signs and symptoms [16,17]. Blood films were prepared for malaria parasite examination, and the packed cell volume (PCV) was measured if there was a history of fever in the preceding 24 hrs, the axillary temperature was ≥ 37.5°C or the child appeared pale. In Ifakara a previous study showed that prophylactic iron supplements had a significant impact in reducing severe anemia without increasing the risk of malaria [8]. Therefore, iron supplements were dispensed to all children for 4 months (from 2–6 months of age). Iron supplements were not given as part of the Manhiça trial.

### Laboratory methods

Thick and thin blood films were stained and read according to standard, quality-controlled procedures [19]. The packed cell volume (PCV) was measured in a microcapillary tube after centrifugation.

### Efficacy assessment

The impact of IPTi with SP on malaria morbidity was assessed through passive clinical surveillance and cross-sectional surveys carried out at ages12 and 18 months in Ifakara, and at ages 12 and 24 months in Manhiça.

### Effects of IPTi

In the Ifakara study, first or only episodes of clinical malaria were reduced by 59% (95% CI, 41; 72%), and the incidence of severe anemia dropped by 50% (95% CI, 8; 73%) [4]. After the intervention was discontinued, a protective effect of 36% (95% CI, 11; 53%) in the incidence of clinical malaria episodes was observed during the extended follow-up that covered the time at-risk starting 1 month after dose 3 of IPTi until age 2 years [18]. In Manhiça, first or only episodes of clinical malaria were reduced by 22.2% (95% CI, 3.7; 37.0; p = 0.020) The incidence of severe anemia up to age 1 year did not differ significantly between the SP and placebo groups [12.7% (95% CI, -17.3; 35.1%), p = 0.36]. The incidence of clinical malaria did not differ between the two intervention arms after discontinuation of IPTi during the extended follow-up. At both sites IPTi was associated with a significant reduction in risk of admission to hospital [Ifakara 28% (95% CI, 7; 45%), Manhiça 19% (95% CI, 4; 31%)].

### Statistical methods and definitions

The original data was re-analyzed at individual level using more stringent criteria than originally to include children in both cohorts that: i) received three doses of IPTi; ii) received the last IPTi dose between 9 and 10 months of age; and iii) had at least 1 month of follow-up after the last IPTi dose. Five periods were defined: i) 1 month after IPTi dose 1; ii) 1 month after IPTi dose 2; iii) 1 month after dose 2 until dose 3; iv) 1 month after dose 3; and v) 6 months, starting 1 month after dose 3.

Clinical malaria was defined as fever (axillary temperature ≥ 37.5°C) plus a *Plasmodium falciparum *asexual parasitaemia. Cox regression models were used to compare the hazards for the time to first or only episode of clinical malaria in each period. The interaction between study and treatment effect was evaluated using the likelihood ratio test.

## Results

Main characteristics of both trials are summarized in Table 1. The most striking difference between the two sites is ITN use. In Ifakara, a successful social marketing scheme promoting ITN use resulted in a 68% ITN coverage [20]. At the time of the trial, the use of ITNs was zero in Manhiça. The HIV seroprevalence was higher in Manhiça (19%) than in Ifakara (6.7%). SP efficacy was similar in the two sites.

**Table 1:** Main characteristics of the area and intervention in Ifakara and Manhiça

	**Ifakara**	**Manhiça**
**Years of intervention**	1999–2001	2002–2004
**Number of children randomized**	701	1503
**Incidence of malaria in placebo group^a^**	0.36	0.43
EIR*	29	38
**Transmission**	Perennial	Perennial with marked seasonality
IPTi regimen**	2, 3, 9	3, 4, 9
Iron supplementation**	2–6	-
**1st line Malaria Treatment during study**	SP	SP + AQ
**ITN use**	68%	0
**HIV seroprevalence (at ANC)**	6.7%	19%
Hb AS prevalence ***	12%	0
**SP parasitological sensitivity#**	69% (62/90)	78.6% (55/70)
**SP ACR#**	94% (84/89)	83% (67/81)

*EIR = Entomological inoculation rate

**months of age

*** hemoglobin AS genotype

# at day 14. ACR = adequate clinical response

^a ^episode/child/year

The analysis estimated the protective efficacy of SP for 30 days after each IPTi dose and found protection above 50% in both sites (Table 2). The analysis also estimated the risk of malaria for the periods when there should have been no pharmacological effect the period between the second and the third IPTi doses and the 6 months beginning 30 days after the third dose (Table 2). The results show that SP efficacy was lower in Manhiça compared to Ifakara between the second and third doses [14.7% (95% CI, -14.6; 36.5) vs 52% (95% CI, 14.0; 74.1)] and for the period after the third dose [7.4 (95%, CI -21; 29.3) vs 32.2% (95% CI, -3.5; 55.6)].

**Table 2:** SP effect on the incidence of clinical malaria during different periods in Ifakara and Manhiça

**Outcome**	**Placebo**			SP*			**Protective efficacy**	**p**
				
	**Events**	**PYAR**	**Rate**	**Events**	**PYAR**	**Rate**	**(95% CI)**	
**During 1 month after dose 1**								
Ifakara	8	21.71	0.37	1	22.21	0.05	87.8% (2.3;98.5)	0.012
Manhiça	13	47.48	0.27	1	49.97	0.02	92.7% (44.2;99.0)	< 0.001
Combined effect adjusted by study	21	69.19	0.30	2	72.18	0.03	90.9% (61.0;97.9)	< 0.001

**During 1 month after dose 2 **								
Ifakara	6	21.83	0.27	0	22.41	0.00	100% (.;100)	0.004
Manhiça	23	47.33	0.49	10	50.27	0.20	59.0% (13.8;80.5)	0.014

**From 1 month after dose 2 until dose 3**								
Ifakara	32	100.08	0.32	16	106.04	0.15	52.8% (14.0;74.1)	0.011
Manhiça	92	169.09	0.54	85	183.36	0.46	14.7% (-14.6;36.5)	0.292

**During 1 month after dose 3**								
Ifakara	14	21.51	0.65	2	22.32	0.09	86.2% (39.4;96.9)	0.001
Manhiça	32	46.94	0.68	16	49.82	0.32	52.7% (13.9;74.1)	0.012

**During 6 months from 1 month after dose 3**								
Ifakara	51	112.56	0.45	37	120.90	0.31	32.2% (-3.5;55.6)	0.070
Manhiça	107	228.31	0.47	104	239.31	0.43	7.4% (-21.3;29.3)	0.578

*Sulphadoxine-pyrimethamine

## Discussion

Intermittent preventive treatment with SP had different protective efficacies in reducing malaria and anemia incidence in these two malaria endemic settings in sub-Saharan Africa. The two trials were very similar in most aspects. Randomization, intervention, follow-up and assessment of outcome procedures were close to identical. The active drug and placebo were manufactured by the same pharmaceutical company. Intensity of malaria transmission during the course of both studies was comparable, not only in the comparable EIR, but also in the rates of malaria episodes in the placebo groups during the first year of life (Table 1). At the time of the trials, parasitological resistance and clinical response to SP were similar, and information obtained shortly before the trials started showed that the drug had an adequate level of efficacy in both settings [12-15]. Moreover, the analysis during the month after each dose (Table 2) shows that SP efficacy was above 50% in both sites. Therefore, it is unlikely that a reduced parasitological efficacy of SP in Manhiça could solely account for the observed difference in clinical protection.

The overall incidence of severe anaemia up to 1 year of age was not significantly affected by the SP intervention in Manhiça [6]. Prophylactic iron supplements were not given to study infants in the Mozambican study since this is not part of the standard of care. In Ifakara, on the other hand, prophylactic ferrous sulphate was given for 4 months to all study participants. The discrepancy in anemia prevention between the two trials could be because malaria is less important than other causes of anaemia in infants in the Manhiça area. An alternative explanation is that protection against malaria needs to be sustained to have a significant impact on associated anaemia. This would also explain the significant effect of IPTi on anaemia in Ifakara where the intervention was given in the context of high ITN's coverage. Moreover, another study in this same setting found a significant reduction in the risk of anaemia in infancy by weekly malaria chemoprophylaxis for 10 months [8].

The administration of the IPTi regimen differed slightly in the two trials because of the different EPI immunization schedules. Consequently, in Ifakara the first two doses were given at 2 and 3 months, while in Manhiça they were administered at 3 and 4 months of age. It is unlikely that this variation in the intervention regimen would explain the difference found in the efficacy; particularly since the time between administration of the second and third dose was shorter in Mozambique. The risk of malaria is greater between age 5 and 9 months than during the first 3 months of life, and thus implies a theoretical advantage to the infants in the Manhiça study, which was not observed [21].

HIV infection during pregnancy has been associated with increased susceptibility to malaria and lower efficacy of IPT with SP [22]. In contrast, no information exists on the impact of the co-infection in children [23]. The HIV infection status was not recorded for children participating in either trial. However, HIV seroprevalence at antenatal clinics differed between the two sites, and it is expected that slightly more enrolled infants in Manhiça would have been infected than in Ifakara. Although specific studies are needed to look at this interaction in children, it is unlikely that HIV associated immunosuppression played an important role in reducing the impact of the intervention in the Manhiça study at the magnitude observed.

The analysis presented in this paper shows that, as expected, the risk of malaria was greatly reduced for 1 month in children who received a stat dose of a moderately effective antimalarial. However, the different protection levels between the two trials is mainly explained by the difference in risk of malaria outside the window of time when the drug is efficacious. There are no obvious reasons that explain these findings. In a previous report we have hypothesized that this reduced risk is a function of the accelerated acquisition of immunity in children receiving IPTi in the first year of life [18]. The question arises why this accelerated acquisition of immunity did not occur in Manhiça. The only remarkable difference between the two sites that could explain this difference refers to the wide-scale use of ITNs in Ifakara. It points to a possible synergistic effect between IPTi and ITNs that reduces the dose of infection from the mosquito and allows an improved acquisition of immunity. This would explain why there is no rebound effect after stopping IPTi in Ifakara, and there is even a sustained reduction of the risk of clinical malaria extending beyond the pharmacological effect of the drug [18]. This additive protection of drugs and ITNs is supported by the results of a study in The Gambia, where the combination of weekly chemoprophylaxis and ITNs provided substantial additional protection against malaria infection and clinical malaria attacks [3].

## Conclusion

This comparative analysis provides an example of how existing data sources can be used to fine-tune malaria control strategies in the immediate future. Contrary to earlier speculation [24], the results suggest that, ITNs and IPTi may act synergistically. Combining these two cost-effective interventions may enhance malaria control, and therefore policy makers considering IPTi as an strategy should include provisions for the sustained use of ITNs. This effect, although encouraging, should be further evaluated in current IPTi trials and future studies, before it can be considered conclusive. Finally, this analysis reiterates the need to carry out careful evaluation of malaria control measures in varied settings before widespread recommendations are made.

## Authors' contributions

*CM*. I declare that I have participated in the conception, design and conduction of the studies, and writing up of the manuscript, and have seen and approved the final version of the manuscript.

*DS*. I declare that I have participated in the conception, design and conduction of the study (Ifakara), and writing up of the manuscript, and have seen and approved the final version of the manuscript.

*EM*. I declare that I have participated in the conduction and analysis of the study (Manhiça), and writing up of the manuscript, and have seen and approved the final version of the manuscript.

*PA*. I declare that I have participated in the conduction of the study (Manhiça), and writing up of the manuscript, and have seen and approved the final version of the manuscript.

*EK*. I declare that I have participated in the conduction of the study (Ifakara), and writing up of the manuscript, and have seen and approved the final version of the manuscript.

*SS*. I declare that I have participated in the analysis of the study (Manhiça), and writing up of the manuscript, and have seen and approved the final version of the manuscript.

*JJA*. I declare that I have participated in the conception, design, and analysis of the studies, and writing up of the manuscript, and have seen and approved the final version of the manuscript.

*JS*. I declare that I have participated in the conduction of the study (Manhiça), and writing up of the manuscript, and have seen and approved the final version of the manuscript.

*HM*. I declare that I have participated in the conception, and design of the study (Ifakara), and writing up of the manuscript, and have seen and approved the final version of the manuscript.

*MT*. I declare that I have participated in the conception, and design of the study (Ifakara), and writing up of the manuscript, and have seen and approved the final version of the manuscript.

*PLA*. I declare that I have participated in the conception, and design of the studies, and writing up of the manuscript, and have seen and approved the final version of the manuscript
